# Biomarker Signatures in Time-Course Progression of Neuropathic Pain at Spinal Cord Level Based on Bioinformatics and Machine Learning Analysis

**DOI:** 10.3390/biom15091254

**Published:** 2025-08-29

**Authors:** Kexin Li, Ruoxi Wang, He Zhu, Bei Wen, Li Xu, Yuguang Huang

**Affiliations:** Department of Anesthesiology, Peking Union Medical College Hospital, Peking Union Medical College, Chinese Academy of Medical Sciences, Beijing 100730, China; b2023001249@pumc.edu.cn (K.L.); wangruoxi@pumch.cn (R.W.); zhuhe166959@student.pumc.edu.cn (H.Z.); wenbei@pumch.cn (B.W.)

**Keywords:** RNA sequencing, neuropathic pain, spared nerve injury, spinal cord, WGNCA, machine learning, time course program

## Abstract

Neuropathic pain (NP) is a debilitating chronic pain condition with complex molecular mechanisms and inadequate therapeutic solutions. This study aims to identify temporal transcriptomic changes in NP using multiple bioinformatics and machine learning algorithms. A total of 10 mouse samples (5 per group) were harvested at each time point (day three, day seven, and day fourteen), following spared nerve injury and a sham operation. Differentially expressed gene (DEG) analysis and an intersection among the three time-point groups revealed 54 common DEGs. The GO and KEGG analyses mainly showed enrichment in terms of immune response, cell migration, and signal transduction functions. In addition, the interaction of the LASSO, RF, and SVM-RFE machine learning models on 54 DEGs resulted in *Ngfr* and *Ankrd1*. The cyan module in WGCNA was selected for a time-dependent upward trend in gene expression. Then, 172 genes with time-series signatures were integrated with 54 DEGs, resulting in 11 shared DEGs. Quantitative RT-PCR validated the temporal expressions of the above genes, most of which have not been reported yet. Additionally, immune infiltration analysis revealed significant positive correlations between monocyte abundance and the identified genes. The TF-mRNA-miRNA network and drug-target network revealed potential therapeutic drugs and posttranscriptional regulatory mechanisms. In conclusion, this study explores genes with time-series signatures as biomarkers in the development and maintenance of NP, potentially revealing novel targets for analgesics.

## 1. Introduction

Neuropathic pain (NP), characterized by spontaneous pain, hyperalgesia, and allodynia [1], is triggered by a somatosensory system injury or dysfunction relevant to primary sensory neurons, the spinal cord, or the brain [2]. The corresponding etiological mechanisms involve mechanical trauma, metabolic disorders, neurotoxicity, infections, malignancies, or stroke [3].

The estimated global prevalence of pathological pain ranges between 6.9% and 10% [4], and approximately 20% of them meet the diagnostic criteria for NP [5]. NP induces both physical and psychological stress in patients, requires long-term management, and incurs elevated treatment costs, thereby imposing a substantial economic and social burden. Major challenges in current NP treatment include suboptimal therapeutic efficacy and drug-related adverse effects, notably respiratory depression, tolerance, and addiction [6,7]. These challenges arise primarily from its complex pathophysiology, refractory nature, and molecular mechanisms.

The spared nerve injury (SNI) model is a widely used experimental model for inducing trauma-related NP through injury to the tibial and common peroneal nerves. The SNI model has proven to be a valuable tool for elucidating the molecular mechanisms underlying NP development and maintenance, given that this model reliably recapitulates the pathophysiological features of clinical NP disorders [8,9].

RNA sequencing has emerged as a powerful technology for transcriptome-wide profiling, precisely quantifying the dynamic expression patterns of coding mRNAs and non-coding RNAs (lncRNAs and miRNAs) across diverse biological conditions. The integration of multiple bioinformatic analyses and machine learning algorithms has recently arisen as a popular paradigm in bioinformatic research and offers multi-dimensional analytical perspectives. So far, most investigations have focused on a single time-point after injury, which only provides a transient glimpse into the dynamic molecular alterations of NP. Dai et al. [10] recently underscored the importance of considering temporal changes on the transcriptome (e.g., circadian rhythms). Elucidating time-series changes in transcriptome profiles in the SNI model may provide insights for potential therapy targets for NP therapeutic strategies.

Therefore, this study utilized RNA sequencing technology to identify potential differentially expressed genes (DEGs) of spinal cord samples across three time points (day three, day seven, and day fourteen) following the SNI procedure. Furthermore, the enriched pathways, three machine learning algorithms, Weighted Gene Co-expression Network Analysis (WGCNA), and immune infiltration analysis, as well as a TF-mRNA-miRNA network and drug-target network, were employed to exhibit genes across different phases of NP and identify temporally patterned biomarkers, seeking phase-specific therapeutics.

## 2. Materials and Methods

### 2.1. Animals and Groups

Six-week-old male C57BL/6J mice (weighing 19−22 g) were procured from SPF Biotechnology (Beijing, China) and group-housed under standard conditions (specific-pathogen free, temperature: 23 ± 2 °C, humidity: 45−50%, under a 12 h/12 h light/dark cycle), with ad libitum access to food and water at the Experimental Animal Center of Peking Union Medical College Hospital (PUMCH; Beijing, China). All animals were habituated to the environment for two weeks before the experiments.

Experimental procedures involving the animals underwent welfare and ethical inspection conducted by the PUMCH Animal Welfare and Ethics Committee (XHDW-2024-113). The animals were randomly assigned to the SNI group and the sham group. The investigators were blinded to the randomized grouping of the animals for all the experiments.

### 2.2. SNI Model

The SNI and sham models were established in accordance with methodologies outlined in previous studies [11]. Briefly, after anesthesia induction with 1.25% bromethol (0.2 mL/10 g i.p., YIHE, Beijing, China), preoperative hair removal was performed, then a 1 cm longitudinal incision was made on the left lateral thigh. The tip of a pair of sterile scissors was used to perform a blunt dissection of the skin and the muscle layer, revealing the sciatic nerve and its three terminal branches (the sural, common peroneal, and tibial nerves) just beneath the muscle. The tibial and common peroneal nerves were tightly ligated with a 6-0 suture, and the sural nerve branch was avoided, followed by sectioning and removing a segment 2 mm distal to the ligation. The muscle layer was closed, and the skin was sutured. For the sham operation, mice underwent only exposure of the sciatic nerve without ligation.

### 2.3. Behavioral Tests

Behavioral tests were performed in a quiet room from 8:00 to 12:00 a.m. Prior to testing, mice were acclimatized in the testing environment for 30 min for two consecutive days and were in the measurement room for 30 min prior to each behavioral test. Mechanical allodynia, spontaneous pain, cold allodynia and thermal hyperalgesia were applied to assess nociceptive behaviors, which were observed 1 day before the operation and on 1, 3, 7, 10, and 14 days postoperatively. Mice were individually placed in acrylic compartments (8 × 12 × 15 cm^3^) with a wire mesh floor. Hind paw mechanical allodynia was measured using a calibrated electronic von Frey apparatus (IITC Life Science, Los Angeles, CA, USA), perpendicularly to the lateral plantar surface of the left hind paw. Simultaneously, spontaneous guarding behavior was scored as 0 (no guarding, paw flat on floor), 1 (mild shift of weight away from paw), 2 (unequal weight bearing and part of the foot not touching the floor), or 3 (foot completely raised or not bearing any weight), shown in Figure S1. These scores were recorded just before each application of the von Frey filament (six observations per paw total and averaged [12]). Cold sensitivity was assessed using the acetone test, as previously described [13]. Briefly, acetone was applied to the plantar surface of the hind paw, and nociceptive responses (including paw withdrawal, licking, or shaking) were scored.

Hind paw thermal hyperalgesia was carried out using the hot-plate apparatus (Cat. No. 35150, Ugo Basile, Emilia-Romagna, Italy), with the temperature set at 55 °C. The time taken to withdraw from the heat stimulus or licking ipsilateral paw was recorded as thermal latency, with a 30 s cutoff time to avoid tissue injury. For all behavioral tests, three repeated measurements were taken for each subject with a 10 min interval, with the average being calculated in this way.

### 2.4. Tisue Collection and RNA Extraction

After euthanasia with 1.25% bromethol and cardiac perfusion with PBS (1×, 4 °C), ipsilateral L1–2 lumbar enlargement of the surgery side (left) was dissected 3, 7, and 14 days post-operatively in an RNase-free environment. The samples were snap-frozen in liquid nitrogen and then frozen/stored at −80 °C until being analyzed. The total RNA of each sample was extracted using a TRIzol Universal kit (No. DP424, TIANGEN, Beijing, China) following the manufacturer’s protocol. The quality and quantity of RNA in each sample were measured using a Nanodrop ND-1000 (NanoDrop, Wilmington, DE, USA). mRNA was purified from total RNA using poly-T oligo-attached magnetic beads. Five mice of each group formed five biological replicates.

### 2.5. cDNA Library Preparation and RNA Sequencing

The cDNA libraries were constructed using Fast RNA-seq Lib Prep Kit V2 (Cat. No. RK20306, ABclonal, Wuhan, China). Qualified libraries were pooled and sequencing was performed using Illumina NovaseqX Plus (Illumina, San Diego, CA, USA). Differential expression analysis for two groups was performed using the limma R package Version 2024.12.1. |log2Foldchange| > 0.585 and *p* < 0.05 were considered as the criteria for evaluating DEGs screened by limma.

### 2.6. Protein–Protein Interaction (PPI) Network Construction

In total, 54 DEGs were uploaded to the STRING database v12.0 (https://cn.string-db.org/, accessed on 8 April 2025). STRING, for functional protein association networks, is employed to identify the predicted protein interactions. A high confidence interaction score of >0.7 was set [14]. The PPI network map of the STRING database was visualized using Cytoscape software (version 3.10.1). In Cytoscape, each gene was considered a node and the connection degree of each protein, namely, the number of proteins it connects, was calculated to evaluate its importance in this network and shown as the degree of color. The significant modules and core interactions were identified using the Cytoscape plugin cytoHubba for discovering densely connected nodes in the network. 

### 2.7. Functional Enrichment Analysis

Gene Ontology (GO) functional enrichment analysis and Kyoto Encyclopedia of Genes and Genomes (KEGG) pathway enrichment analysis on the DEG set were implemented using the clusterProfiler R package. GO analysis has been developed as an efficient way to carry out functional enrichment on a large scale to identify several significantly enriched GO terms across biological processes, cellular components, and molecular functions. KEGG is also an extensively applied database using hypergeometric distribution testing, and it preserves extensive data on drugs, chemical substances, diseases, biological processes, and signaling pathways. *p* < 0.05 was understood to indicate statistical significance when conducting the GO and KEGG analysis.

### 2.8. Machine Learning Algorithms

Three machine learning algorithms (namely, Least Absolute Shrinkage and Selection Operation (LASSO), Random Forest (RF) and Support Vector Machine—Recursive Feature Elimination (SVM-RFE)) were employed to screen characteristic genes. Before the machine learning analysis, identified common core genes with normalized values in various samples were converted into a matrix format to facilitate subsequent model calculations. Specific information was extracted and read using R scripts.

Random forest, a versatile predictive algorithm combining multiple decision trees through ensemble learning to generate robust predictions across diverse conditions, was executed through the “randomForest” package in R with a hyperparameter setting of nTree = 1000. The optimal number of trees (“optionTrees”) was subsequently determined by analyzing the error curve of the model and the model was rebuilt. Although no direct feature selection parameters were configured, the extraction and visualization of feature importance implicitly reflect the feature selection process. The importance score of each gene (feature) was retrieved from the random forest model and converted into a dataframe format for subsequent visualization.

The LASSO machine learning algorithm, which simplifies high-dimensional data by including independent variables with non-zero coefficients, was implemented utilizing the “glmnet” R package. Hyperparameters in LASSO included ‘alpha = 1’ and ‘nlambda = 100’ with 10-fold cross-validation (‘nfolds = 10’) in R. Family = “binomial” was used to specify the type of model; ‘lambda.min’ and ‘lambda.1se’ were used to select the optimal value of lambda to obtain the optimal model while maintaining the stability of the model’s performance.

SVM-RFE, a supervised machine-learning protocol, enhances the discriminative power of biomarkers and was conducted using the “e1071” R package with 5-fold cross-validation. Hyperparameters in SVM-RFE also included ‘k = 5’, ‘halve.above = 100’, ‘Error Rate’ and ‘Accuracy’. These hyperparameters were made to balance efficiency and accuracy in feature selection based on the size of the dataset and the number of features. ‘which.min(errors)’ was used to select the optimal number of features by finding the point with the lowest error rate. Subsequently, the common set of genes was obtained by intersecting the groups of elements screened from above three machine learning algorithms.

### 2.9. WGCNA

The WGCNA algorithm is a typical systems biology algorithm for constructing gene co-expression networks and identifying gene modules related to biological traits. After filtering outlier data, we finally analyzed 17,280 genes across 30 samples using WGCNA at the Novomagic, an online platform for data analysis (https://magic-plus.novogene.com, accessed on 25 April 2025). An appropriate soft threshold power of 3 was selected to construct the Topological Overlap Matrix (TOM), transforming the adjacency matrix into a weighted adjacency matrix. Based on the TOM matrix, hierarchical clustering analysis was conducted to identify key gene modules and core genes. Subsequently, the intersection between the cyan module genes and 54 DEGs revealed 11 common DEGs.

### 2.10. Quantitative Real-Time PCR (qRT-PCR)

Total RNA of ipsilateral L1–2 lumbar enlargement of the surgery side (left) was extracted using an RNAeasyTM Animal RNA isolation kit (Cat. R0027, Beyotime, Beijing, China) and reverse transcribed into cDNA using HiScript IV All-in-One Ultra RT SuperMix (Cat. R433-01, Vazyme, Beijing, China). Quantitative PCRs (qPCRs) were performed using SupRealQ Ultra Hunter SYBR qPCR Master Mix (U+) (Q713-02, Vazyme, Beijing, China). Amplification and melting curves were recorded using a StepOne Real-Time PCR System (ABI, Carlsbad, CA, USA). Gene expression levels were quantified by normalizing against GAPDH and expressed as the fold change. Primers for the selected genes are shown in Table 1.

### 2.11. Immune Infiltration Analysis

The ‘CIBERSORT’ package in R was applied to estimate the difference and proportion of immune infiltration between the SNI and the sham groups in 22 types of immune cells and immune-associated features. Considering the interspecies differences between humans and mice, gene symbols from LM22 in the immune infiltration analysis software were matched with identified core gene symbols, and only genes with exact symbol matches were retained for subsequent analyses. *p* < 0.05 was considered statistically significant. Subsequently, the Spearman correlation analyses between immune cells and core DEGs were performed.

### 2.12. Receiver Operating Characteristic (ROC) Analysis

ROC curves, generated using the ‘pROC’ package in R, were utilized to assess the diagnostic effectiveness of the core genes for NP. These curves delineate the equilibrium between sensitivity and specificity, and the clinical diagnostic value of genes was quantified by calculating the area under the curve (AUC).

### 2.13. Regulatory Network of TF, mRNAs and miRNAs and Identification of Potential Drugs

TF and miRNA information was obtained from two online databases, and then the TF–mRNA–miRNA network was visualized using Cytoscape. TF–target gene interactions were ascertained through Transcriptional Regulatory Relationships Unraveled by Sentence-based Text mining (TRRUST), which contains the targets gene corresponding to the TFs and the regulatory relationships between TFs. Moreover, miRNA–target interactions were predicted using miRWalk 3.0, an openly available online database.

The Drug Gene Interaction Database (DGIdb) (version 5.0, https://www.dgidb.org, accessed on 8 May 2025), an openly accessible database, was employed to present information on drug–gene interactions, which was then visualized utilizing Cytoscape to identify the potential drugs.

### 2.14. Statistical Analysis

Quantitative variables are presented as the means ± standard error of the mean (SEM). Student’s *t*-tests were used to compare differences between two groups, whereas repeated-measures one-way ANOVAs were used for comparisons among three or more groups, followed by Dunnett’s post hoc test for multiple comparisons. Post hoc tests with Dunnett correction were applied for multiple comparisons. A *p* value of <0.05 was regarded as significant. All statistical analyses were conducted using GraphPad Prism 10.4.1 (GraphPad Software, Inc., San Diego, CA, USA).

## 3. Results

### 3.1. Overall Workflow and the Development of Nociception and Cold Hypersensitivity in the SNI Model

**Figure 1:**
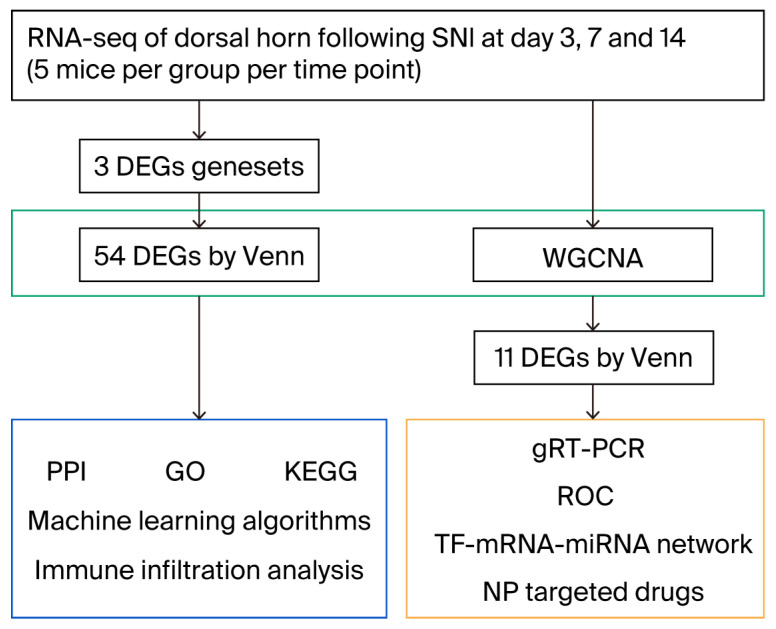
Analysis workflow of the research.

**Figure 2:**
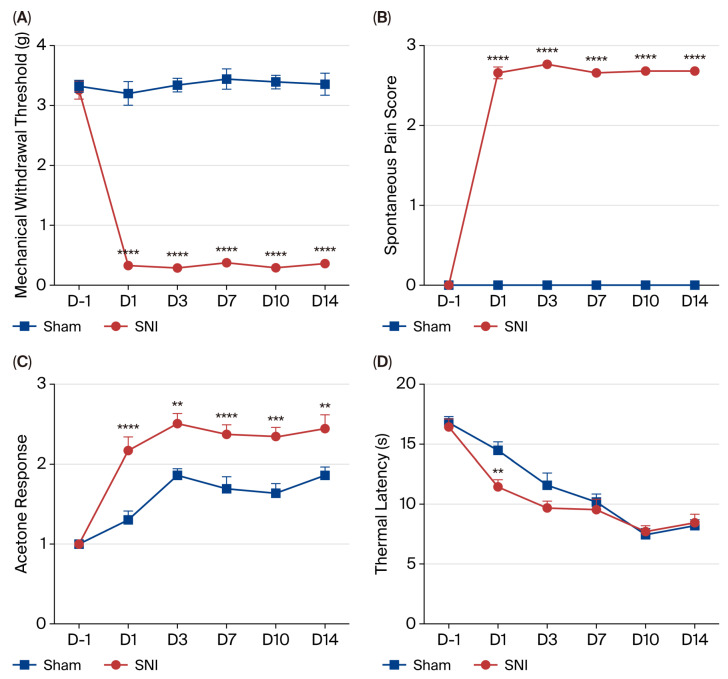
Behavioral changes in mice from SNI and sham groups at multiple time points (D-1, 1, 3, 7, 10 and 14). (**A**) Assessment of mechanical allodynia using the von Frey test, *n* = 12 per group. (**B**) Evaluation of spontaneous pain by spontaneous pain score, *n* = 12 per group. (**C**) Assessment of cold allodynia by the acetone test, *n* = 12 per group. (**D**) Thermal hyperalgesia was assessed using the hot-plate apparatus in SNI and sham groups, *n* = 12 per group. Data are represented as the mean ± SEM. **, *p* < 0.01; ***, *p* < 0.001; ****, *p* < 0.0001.

The complete flowchart of this study is given in Figure 1. To ensure the occurrence of NP, associated behavior tests were assessed on preoperative day 1 and postoperative day 1, day 3, day 7, day 10 and day 14. There was a significant decrease in the mechanical pain threshold (*p* < 0.0001; Figure 2A) and increases in the spontaneous pain score (*p* < 0.0001; Figure 2B) and cold hypersensitivity (*p* < 0.01; Figure 2C) in the SNI group compared to the sham group. Though there was no difference in heat nociception except on postoperative day 1 (*p* < 0.01; Figure 2D), these results suggest that the neuropathic pain model was successfully established in mice with SNI.

### 3.2. Identification of Common DEGs Across Three Time Points

**Figure 3:**
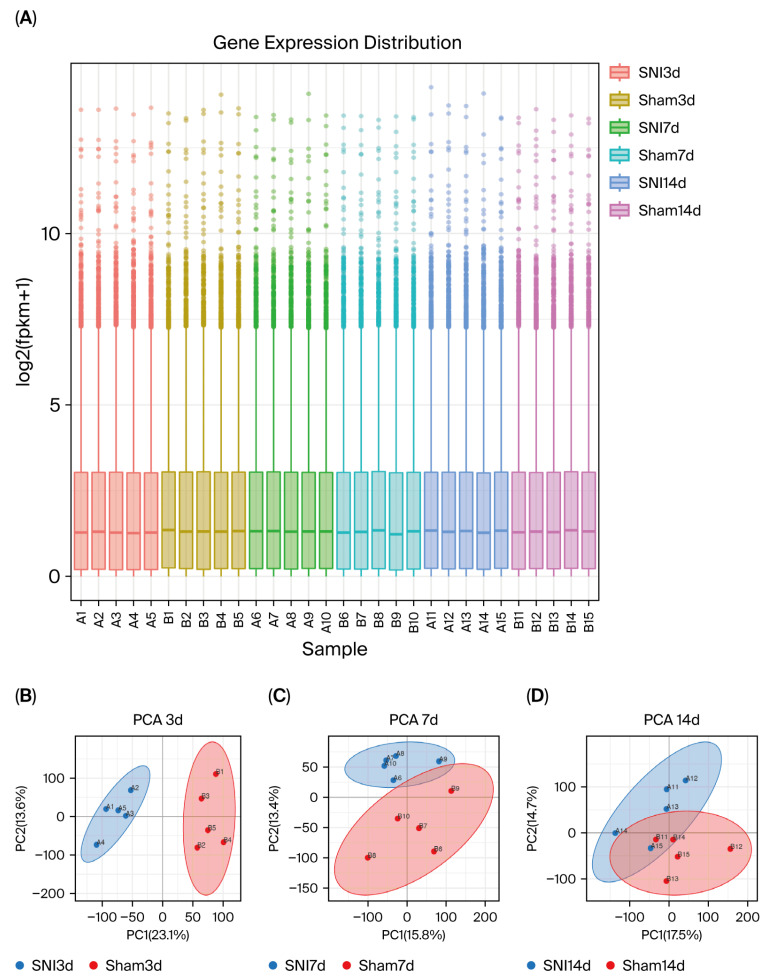
Gene expression distribution following normalization and PCA demonstration at three time-point groups. (**A**) Consistent distribution of expression data across samples of six groups in the boxplot of gene expression distribution. (**B**) PCA plot of gene expression profiles between SNI and sham groups at day 3. (**C**) PCA plot of gene expression profiles between SNI and sham groups at day 7. (**D**) PCA plot of gene expression profiles between SNI and sham groups at day 14. PCA, principal component analysis. PCA plots were plotted using the OmicShare tools, a free online platform for data analysis (www.omicshare.com/tools, accessed on 10 May 2025).

A total of 30 samples were harvested (5 in each group) at day 3, day 7 and day 14 after SNI or sham surgery for RNA sequencing. The gene expression boxplot in Figure 3A illustrates the consistent distribution of expression data across six groups, validating their suitability for analysis. To identify the DEGs at different time points and common DEGs across three time points, differential gene analysis was performed using the limma package with a *p* < 0.05 and |log2Foldchange| > 0.585 as the standard.

**Figure 4:**
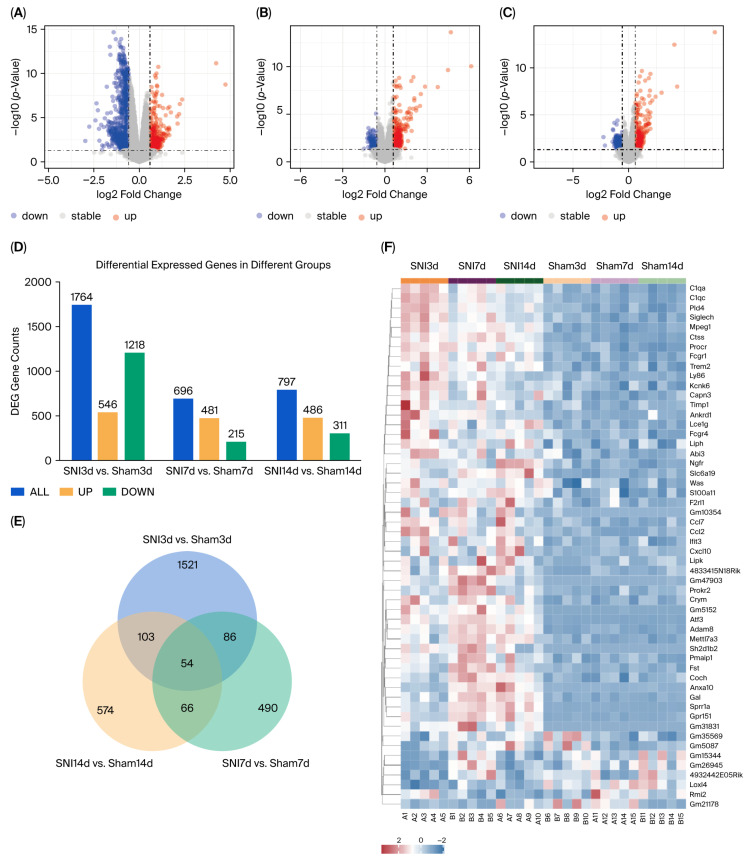
Differential gene analysis and identification of common differentially expressed genes. (**A**–**C**) Volcano plot of differential gene expression at day 3, day 7 and day 14. (**D**) Detailed information on differentially expressed gene counts. (**E**) Venn diagram presenting 54 common differentially expressed genes across day 3, day 7, and day 14. (**F**) Heatmap of hierarchical clustering indicates differential expression signatures of 54 genes between the SNI and sham groups. Venn diagrams and heatmaps were plotted using the OmicShare tools, a free online platform for data analysis (www.omicshare.com/tools, accessed on 10 May 2025).

The principal component analysis (PCA) of the respective groups is shown in Figure 3B–D. The results revealed a total of 1764 DEGs (546 upregulated and 1218 downregulated) at day 3 (Figure 4A), 696 DEGs (481 upregulated and 215 downregulated) at day 7 (Figure 4B), and 797 DEGs (486 upregulated and 311 downregulated) at day 14 (Figure 4C) in the SNI group compared to the sham group (Figure 4D). Furthermore, the intersection of DEGs for day 3, day 7 and day 14 yielded 54 shared DEGs (Figure 4E) of significant interest, and the detailed information is given in Supplementary Table S1. Simultaneously, we present a heatmap depicting the expression patterns of the 54 identified DEGs (Figure 4F).

### 3.3. PPI Analysis of 54 DEGs and Hub Genes

**Figure 5:**
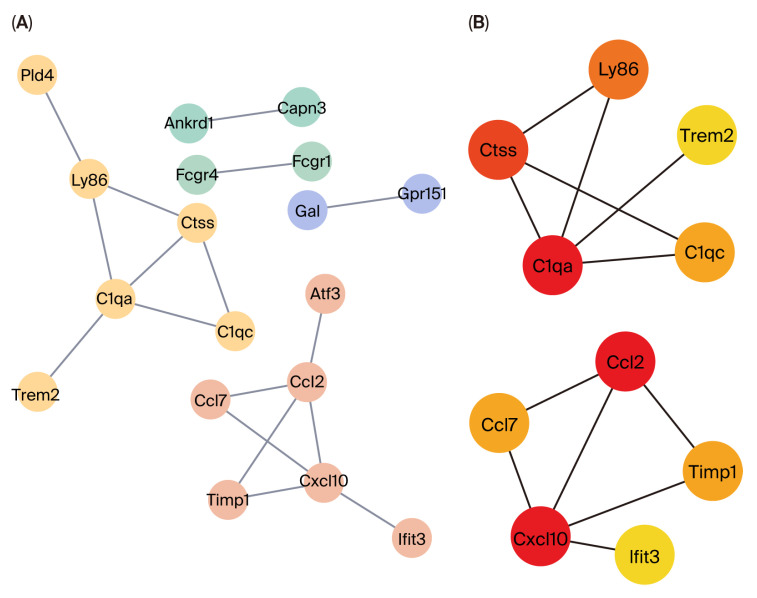
PPI network and hub gene identification based on 54 DEGs. (**A**) The PPI network analysis of 54 DEGs. (**B**) Identification of 10 hub genes. Nodes in darker colors represent a higher degree. PPI, protein–protein interactions; DEGs, differentially expressed genes.

The PPI network was constructed to explore intricate interactions among these 54 DEGs via the STRING database with high confidence set at 0.7 (Figure 5A). The network consists of 45 nodes (proteins) and 17 connecting edges. The top 10 hub genes (*Ccl2*, *Cxcl10*, *C1qa*, *Ctss*, *Ly86*, *Timp1*, *Ccl7*, *C1qc*, *Ifit3*, *Trem2*) differentially expressed throughout the acute and chronic stage of NP were screened out using cytoHubba in Cytoscape software (Figure 5B). Additionally, we drew PPI networks of three distinct time points to show the top 10 hub genes in different stages of NP (Supplementary Figure S2).

### 3.4. GO and KEGG Enrichment Analysis of 54 DEGs

**Figure 6:**
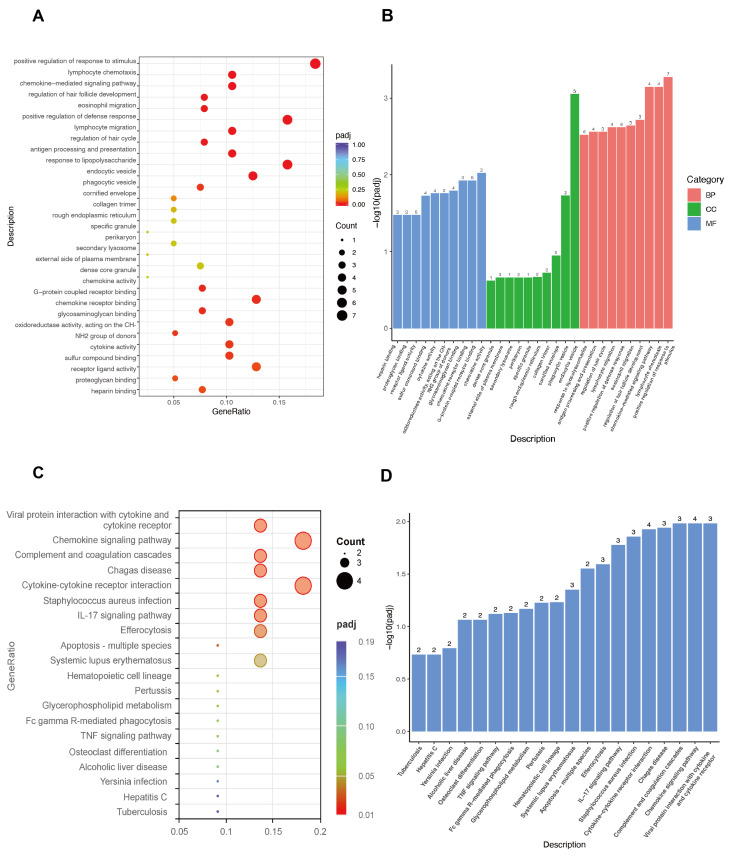
Top 10 items of the function enrichment analysis of the gene set across three time points. (**A**,**B**) Gene ontology analysis, including biological processes (BP), cellular components (CC), and molecular functions (MF). (**C**,**D**) Kyoto Encyclopedia of Genes and Genomes analysis.

The GO functional annotation and KEGG pathway enrichment analysis were applied based on the 54 DEGs with *p* < 0.05. As illustrated in Figure 6A,B, the GO enrichment highlighted the biological processes primarily associated with the positive regulation of responses to external stimulus, lymphocyte chemotaxis, and the chemokine-mediated signaling pathway, followed by eosinophil migration, positive regulation of defense response, lymphocyte migration, and antigen processing and presentation (Supplementary Figure S3). Regarding cellular components (Supplementary Figure S4), enrichment was observed in the endocytic vesicle and phagocytic vesicle, while molecular functions were predominantly enriched in chemokine activity, G-protein-coupled receptor binding, chemokine receptor binding, glycosaminoglycan binding, cytokine activity, sulfur compound binding, receptor ligand activity, and cytokine receptor binding (Supplementary Figure S5). KEGG enrichment analysis revealed that these 54 DEGs are strongly related to viral protein interactions with cytokine and cytokine receptors, the chemokine signaling pathway, complement and coagulation cascades, and cytokine–cytokine receptor interactions (Figure 6C,D). Additional details can be found in Supplementary Table S2. To summarize, these findings provide a solid theoretical foundation for a deeper exploration of immune response, cell migration, and signal transduction in the pathogenesis of NP.

### 3.5. Identification of Characteristic Genes via Machine Learning Algorithms

**Figure 7:**
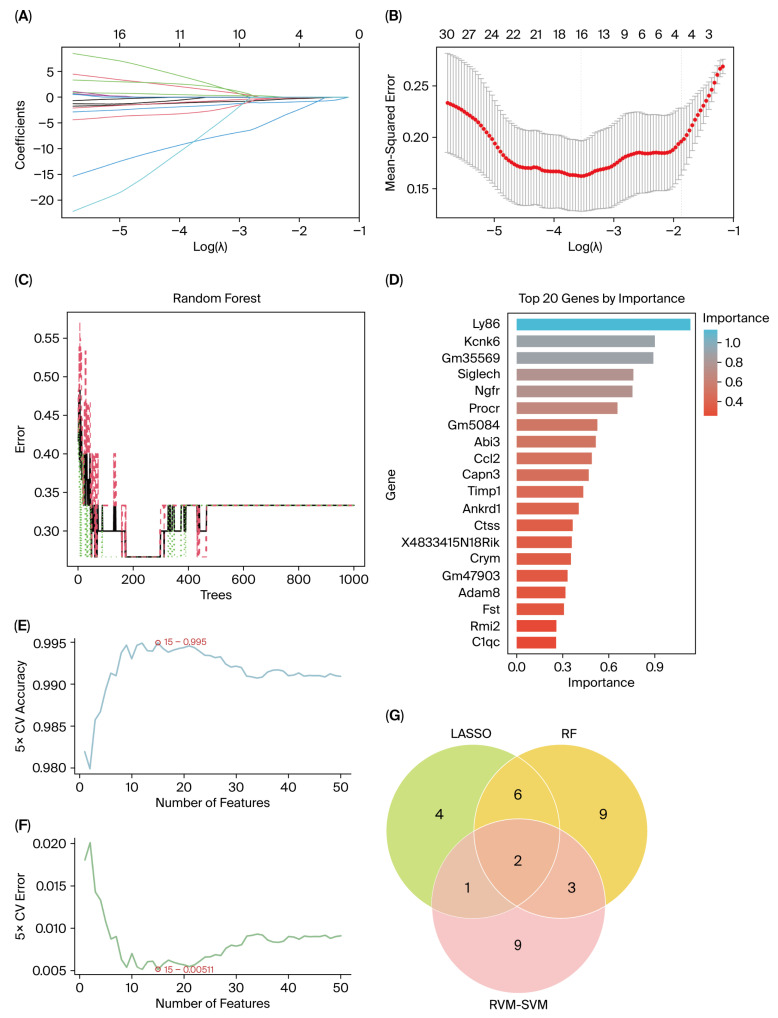
Machine learning algorithms on 54 DEGs. (**A**,**B**) The 13 candidate genes via LASSO regression with 10-fold cross-validation. (**C**,**D**) Identification of top 20 genes by RF algorithms. (**E**,**F**) Detection of 15 characteristic genes in the SVM-RFE algorithm. (**G**) Two common genes identified through the interaction of three machine learning algorithms: *Ngfr* and *Ankrd1*. DEGs, differentially expressed genes; LASSO, Least Absolute Shrinkage and Selection Operation; RF, Random Forest; SVM-RFE, Support Vector Machine—Recursive Feature Elimination. The Venn diagram was plotted using OmicShare tools, a free online platform for data analysis (www.omicshare.com/tools, accessed on 10 May 2025).

As shown in Figure 7A,B, 13 DEGs were identified in the LASSO logistic regression, including *Siglech*, *Ly86*, *Ngfr*, *Kcnk6*, *Lce1g*, *Abi3*, *Cxcl10*, *Slc6a19*, *Gm35569*, *Ankrd1*, *Prokr2*, *Lipk* and *Gm5084* (detailed information given in Supplementary Table S3). Analysis of the RF algorithm is displayed in Figure 7C. Then, 20 key genes (*Ly86*, *Kcnk6*, *Gm35569*, *Siglech*, *Ngfr*, *Procr*, *Gm5084*, *Abi3*, *Ccl2*, *Capn3*, *Timp1*, *Ankrd1, Ctss*, *X4833415N18Rik*, *Crym*, *Gm47903*, *Adam8*, *Fst*, *Rmi2* and *C1qc*) were selected according to the highest MeanReducedGini values (Figure 7D, Supplementary Table S4). SVM-RFE machine learning algorithms identified 15 genes (*Gm31831*, *Atf3*, *Ctss*, *Gpr151*, *Ankrd1*, *Adam8*, *Trem2*, *Lce1g*, *Mpeg1*, *C1qc*, *C1qa*, *Ngfr*, *Sprr1a*, *Pld4*, and *Sh2d1b2*), which were characterized by the minimal error rate of 0.005 and maximal accuracy of 0.995 (Figure 7E,F; Supplementary Table S5). Subsequently, the intersection of above three sets of genes screened out two genes displayed in the Venn diagram (Figure 7G), namely, *Ngfr* and *Ankrd1*.

### 3.6. Selection of Modules with Time-Course Progression by WGCNA

**Figure 8:**
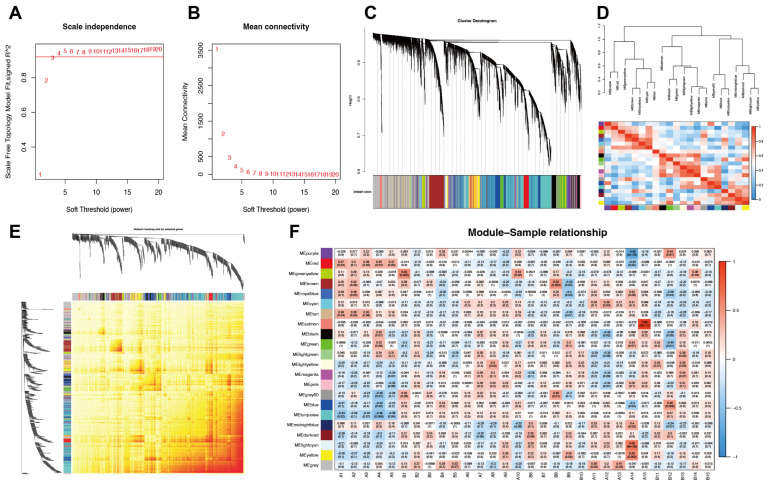

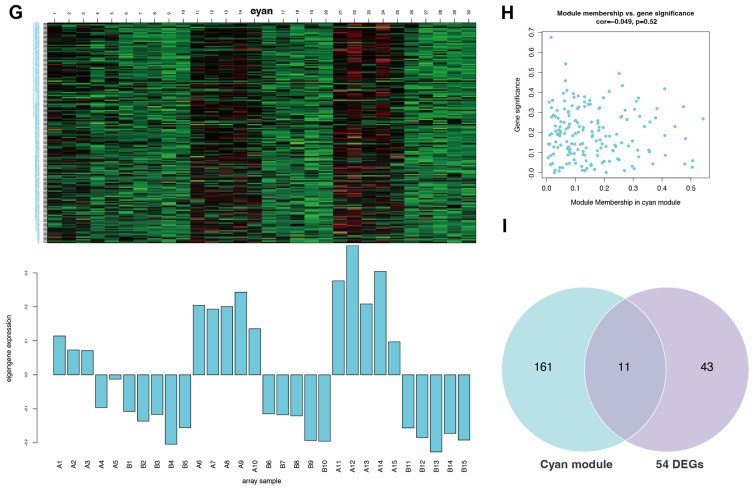
Identification of key modules with temporal characteristics. (**A**,**B**) The selection of the optimal soft-threshold power as 3 (R^2^ = 0.9). (**C**) Gene clustering dendrogram. (**D**) Heatmap of the association among modules. (**E**) Network heatmap plot for selected genes. (**F**) Heatmap of module–sample relationships. (**G**) Heatmap of eigengene expression in the cyan module. (**H**) Scatter plots of the cyan module illustrating the relationship between gene significance and module membership in the cyan moule. (**I**) Venn diagram between cyan module and 54 DEGs. Eleven common DEGs were selected. DEGs, differentially expressed genes. The Venn diagram was plotted using OmicShare tools, a free online platform for data analysis (www.omicshare.com/tools, accessed on 10 May 2025).

All samples were included in the WGCNA, and outlier data were filtered. Based on scale independence and mean connectivity (Figure 8A,B), the scale-free network criteria (R^2^ = 0.9) already met at a lower soft-thresholding power (β = 3), indicating strong co-expression patterns among genes. Since the ultimate purpose of WGCNA is to identify biologically meaningful modules, a β = 3 has already balanced the scale-freeness and the retention of network information (avoiding over-sparse networks); therefore, power = 3 was finally determined. In total, 21 co-expression modules were identified utilizing the dynamic tree-cut method and they are illustrated in heatmap of module–trait relationships (Figure 8C,D), in which each color represents a co-expression module with module–sample correlation coefficients and *p*-values (Figure 8E,F). The cyan module containing 172 genes was selected for an upward trend of expression over time (Figure 8G,H), which was subsequently integrated with 54 DEGs and identified 11 common genes, namely, *S100a11*, *Gal*, *Ifit3*, *Ngfr*, *Coch*, *Pmaip1*, *Adam8*, *Mettl7a3*, *Fst*, *Cxcl10* and *Slc6a19* (Figure 8I).

### 3.7. Validation of Bioinformatics Results by qRT-PCR

**Figure 9:**
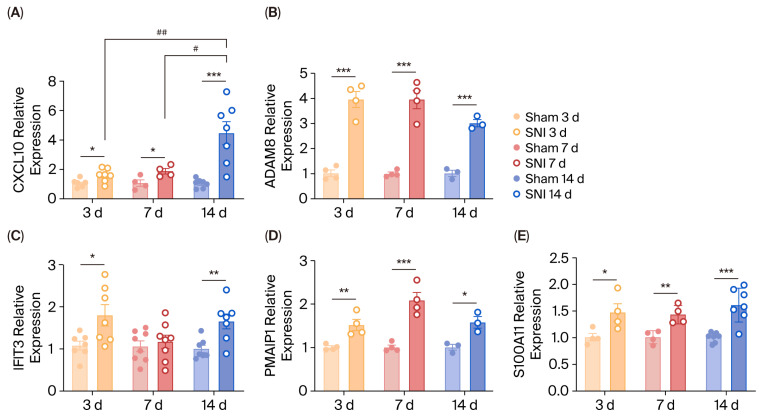
Validation of DEG expression by qRT-PCR. (**A**–**E**) Temporal changes in mRNA expression levels of *Cxcl10*, *Adam8*, *Ifit3*, *Pmaip1* and *S100A11*, *n* = 4–8 per group. Data are represented as mean  ±  SEM. * represents a comparison between SNI and sham at the same time point, while ^#^ represents a comparison between SNI groups at various time points. *, *p* < 0.05; **, *p*  <  0.01; ***, *p*  <  0.001; ^#^, *p* < 0.05; ^##^, *p* < 0.01. DEGs, differentially expressed genes; qRT-PCR, quantitative real-time PCR.

qRT-PCR analysis was applied to detect the mRNA expression levels of *Cxcl10*, *Adam8*, *Pmaip1*, *S100a11* and *Ifit3* that were not previously verified in the NP among 11 DEGs (Figure 9). Compared with the sham group, the expression levels of *Cxcl10*, *Adam8*, *Pmaip1* and *S100a11* were upregulated at day 3, day 7 and day 14 following SNI (*p* < 0.05, Figure 9A,B,D,E). For *Ifit3*, there was a higher expression level in the SNI group at day 3 (*p* < 0.05) and day 14 (*p* < 0.01, Figure 9C).

From the perspective of the progression of NP, *Cxcl10* exhibited an overall significant upward trend (Figure 9A), with differences between day 3 and day 14 (*p* < 0.01), as well as day 7 and day 14 (*p* < 0.05). In addition, *Adam8*, *Ifit3* and *S100a11* displayed comparable expression levels at day 3, day 7, and day 14 (Figure 9B,C,E). Pmaip1 seemed to have a slight fluctuation over time with a brief and insignificant rise at day 7 relative to day 3 and day 14 (Figure 9D).

Some unproved genes of interest in the rest of the 54 DEGs were also validated and show evaluated expression levels in the SNI group at day 3, day 7, and day 14 (Supplementary Figure S6), except *Cd180* at day 14 (Supplementary Figure S6E). *Siglech*, *Mpeg1*, *Ly86*, *Crym*, *Cd180*, *Procr* and *Abi3* experienced gradual downregulation with statistical differences (Supplementary Figure S6). For *Procr*, there was significant difference among day 3, day 7 and day 14 (*p* < 0.05, Supplementary Figure S6F). A significant decrease in *Abi3* was observed between day 3 and day 14 (*p* < 0.001), as well as between day 7 and day 14 (*p* < 0.01, Supplementary Figure S6G). As for the others, the most notable reduction was only observed between day 3 and day 14 (*p* < 0.05) in Supplementary Figure S6A–E. *Pld4* displayed comparable expression levels at day 3, day 7 and day 14 (Supplementary Figure S6H). The expression profiles quantified via qRT-PCR demonstrated strong concordance with the bioinformatic predictions.

### 3.8. Immune Characteristics of SNI Model

**Figure 10:**
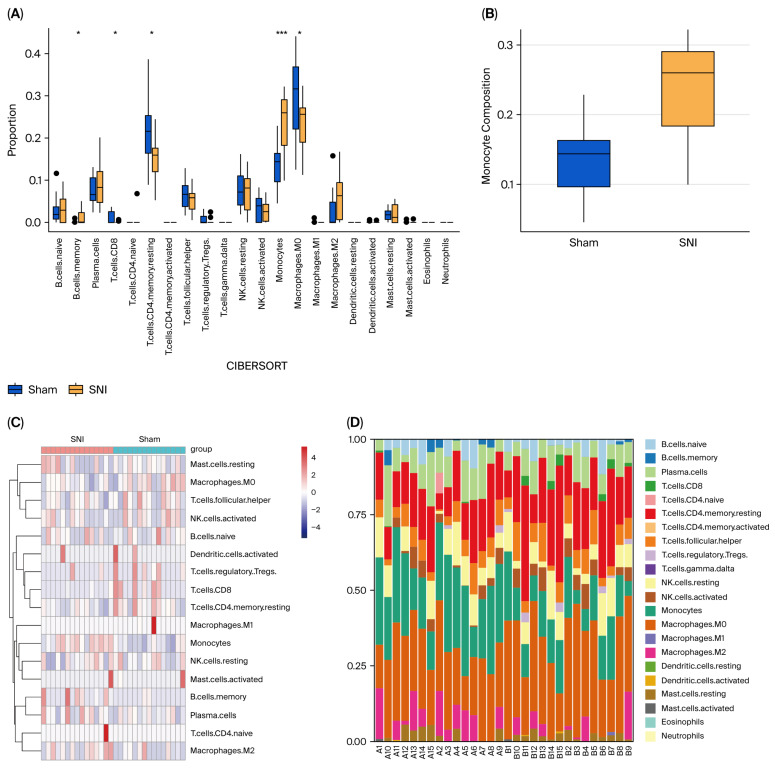

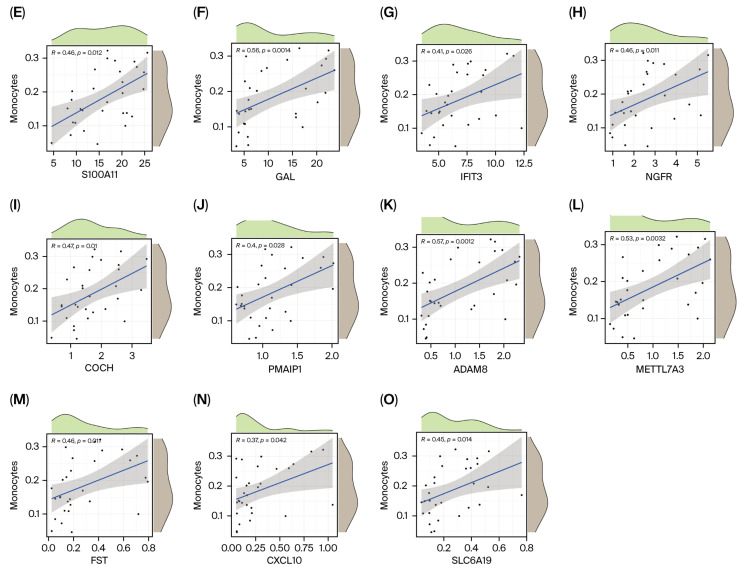
Evaluation of the immune cell infiltration between the sham group and SNI groups. (**A**) Infiltration of 22 immune cells in the sham group and SNI group. *, *p* < 0.05; ***, *p* < 0.001. (**B**) Significance comparison of monocyte composition between the SNI and sham groups. (**C**) The immune infiltration landscape in the SNI and sham groups is demonstrated in the heatmap plot. (**D**) Stacked histogram of changes in the proportion of immune cells. (**E**–**O**) Spearman correlation analysis between monocytes and 11 shared DEGs. SNI, spared nerve injury; DEGs, differentially expressed genes.

**Figure 11:**
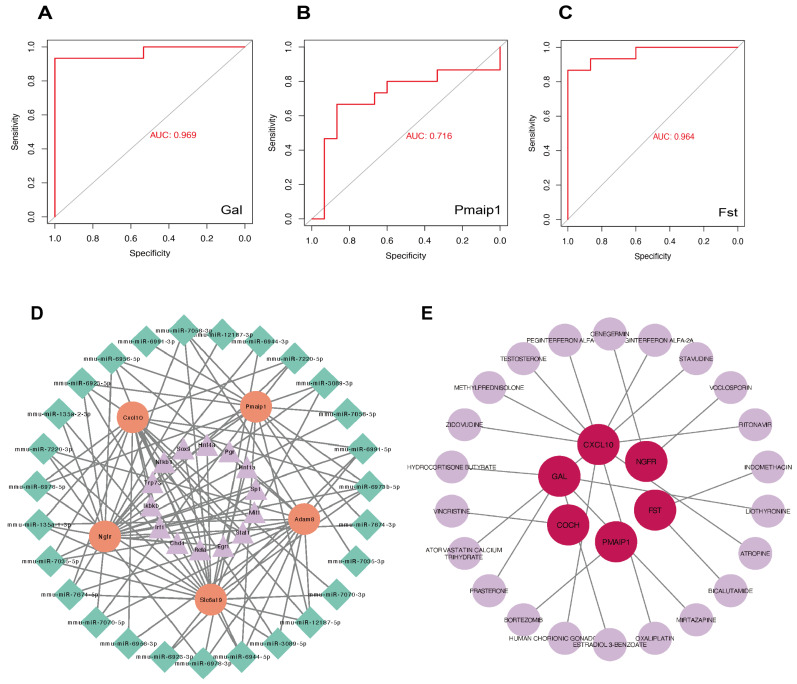
Diagnostic values, TF–mRNA–miRNA network construction and prediction of 11 DEG-targeted drugs. (**A**–**C**) ROC curves showing there were three genes with significant diagnostic value (AUC > 0.7): *Gal*, *Pmaip1* and *Fst*. (**D**) In total, 14 TFs and 14 miRNAs are included based on 11 DEGs. Outer green rhombuses represent selected miRNA. Orange circles represent mRNAs. Purple triangles mean predicted TFs. (**E**) Network diagrams showing that final six gene-related drugs on the DGIdb database. The dark purple nodes represent genes, and the shallow purple nodes show targeted drugs. ROC, receiver operating characteristic; DEGs, differentially expressed genes; AUC, area under the curve. TF, transcription factor.

A comparative analysis of immune cell infiltration between the SNI group and sham group was conducted based on 54 DEGs (Figure 10). The SNI group exhibited significantly upregulated infiltration of B cell memory (Figure 10A) and monocytes (Figure 10A,B). Conversely, there was a notable proportion of T cells CD8, T cells CD4 (memory resting), and macrophages M0 in the sham groups (Figure 10A). The immune infiltration landscape and comparative distribution of immune cells in the SNI and sham groups are illustrated in Figure 11C,D. Since monocytes have a dominant percentage of infiltration in the SNI group compared to the sham group (Figure 10B), a correlation analysis between monocytes and the 11 DEGs was subsequently performed, exhibiting a positive correlation between monocytes and *S100a11*, *Gal*, *Ifit3*, *Ngfr*, *Coch*, *Pmaip1*, *Adam8*, *Mettl7a3*, *Fst*, *Cxcl10* and *Slc6a19* (*p* < 0.5, Figure 10E–O).

### 3.9. Assessment of the Clinical Diagnostic Value of Genes

An ROC curve analysis was performed on 11 DEGs to evaluate their clinical diagnostic potential. As depicted in Figure 11A–C, the AUC values of *Gal*, *Pmaip1* and *Fst* exceed 0.7, suggesting their significant value in clinical diagnosis. The ROC results of other genes (AUC < 0.7) are shown in Supplementary Figure S7.

### 3.10. TF–mRNA–miRNA Network Construction and NP-Targeted Drug Prediction

The upstream regulation of the 11 DEGs was explored by predicting TFs and miRNAs. We found that 14 TFs were related to five genes, including *Slc6a19*, *Cxcl10*, *Adam8*, *Pamip1* and *Ngfr* (Figure 11D). After predictions were carried out using miRWalk, fourteen miRNAs exhibiting interactions with the target genes were identified and incorporated into the TF–miRNA–mRNA interactions (Figure 11D). Furthermore, we searched potential drugs for 11 DEGs on the DGIdb database. Apart from genes without corresponding drugs, unproved drugs were not exhibited either. Thus, the final gene–drug interaction network consists of six genes: *Gal*, *Ngfr*, *Coch*, *Pmaip1*, *Fst* and *Cxcl10* (Figure 11E). 

## 4. Discussion

Current management of NP remains challenging due to limited efficacy and adverse effects, underscoring an urgent need for improved therapeutic strategies. The transition from acute to chronic phases represents a critical developmental trajectory in NP, with chronic NP posing greater therapeutic challenges. Therefore, exploring temporal changes of transcription profiling on a peripheral nerve injury model might allow us to elucidate mechanisms and identify novel targeted therapies for NP [15]. Three time points representing distinct pathological phases are used in our study to identify the DEGs responsible for the initiation and maintenance of NP.

In this study, we conducted multiple bioinformatics analyses to delve into the full repertoire of genes following SNI across a temporal program. Initially, we identified upregulated genes and downregulated genes in the SNI group in contrast to the sham group at distinct time points. An intersection analysis was performed among the three time points, resulting in 54 common DEGs, which represent our primary interest. Furthermore, GO and KEGG enrichment analyses of these DEGs revealed the significant involvement of immune response, cell migration, and signal transduction in NP pathogenesis, which was further supported by the findings from the immune infiltration analysis. *Ngfr* and *Ankrd1* were identified as characteristic genes through an integrated analysis using LASSO, RF, and SVM-RFE algorithms. WGCNA identified 172 genes in the cyan module. Subsequent analysis prioritized 11 core genes co-expressed between DEGs and WGCNA-derived feature module genes: *Gal*, *Fst*, *Ngfr*, *Coch*, *Mettl7a3*, *Slc6a19*, *S100a11*, *Ifit3*, *Pmaip1*, *Adam8*, and *Cxcl10*. Importantly, most of these genes were identified for the first time.

Among these 11 core genes, *Gal*, *Fst* and *Ngfr* have been extensively studied for their roles in NP pathogenesis. There is accumulating evidence [16,17,18,19] showing the engagement of *Gal* in the NP. *Gal*, the gene encoding galanin, has been identified as a hub gene across different NP models and validated by qRT-PCR [20,21]. Single-cell RNA sequencing was conducted on SNI dorsal root ganglion (DRG) cells, and we identified two SNI-induced neuronal clusters (SNIICs) characterized by *Gal* expression [22], where *Gal* contributes to the SNIIC conversion. *Atf3* and *Egr1* have also been identified as key TFs, the activation of which upregulates *Gal* expression within these clusters. In the dorsal horn, nerve injury induces the upregulation of galanin expression in afferent terminals and is accompanied by enhanced synaptic peptide secretion [23], and galanin exerts the inhibitory modulation of pain processing after nerve injury [24]. FST exerts its biological effects on inhibiting TGF-β superfamily ligands. A-fiber neuron-derived FST was recently shown to potentiate Nav1.7-dependent nociceptive neuronal hyperexcitability through direct interaction with insulin-like growth factor-1 receptor (IGF1R) [25]. Ngfr protein refers to p75 neurotrophin receptor (p75NTR), which produces pain signals by binding to nerve growth factor (NGF) [26] and has been investigated as a therapeutic target in the neuropathic nociception of different NP models [27,28,29,30]. The underlying mechanism may involve Ngfr-mediated topographic nerve regeneration by controlling Schwann cell migratory behavior and myelination processes [31]. However, the functional roles of *Coch*, *Slc6a19* and *Mettl7a3* in pain remain unexplored in the current literature. The studies of *Coch* mainly focus on anxiety-related disorders [32] and autosomal dominant hearing loss [33]. *Slc6a19* in solute carrier family 6 is mostly reported in terms of weight and adiposity [34]. The human methyltransferase-like (METTL) protein family mediates the methylation of nucleic acids, proteins, and small molecules, thus involving multiple cellular processes [35]. Regretfully, the current understanding of *Mettl7a3*, the gene orthologous to human *Mettl7a*, remains limited, while *Mettl7a* is known to be predominantly expressed in urinary and digestive systems as a cancer biomarker [36].

After excluding the above genes, *S100a11*, *Adam8*, *Pmaip1*, *Cxcl10* and *Ifit3* were selected and then verified using qRT-PCR. We further validated *Siglech*, *Mpeg1*, *Ly86*, *Crym*, *Cd180*, *Procr*, *Abi3* and *Pld4* from the remaining 44 DEGs, which are of interest and have not been validated, in order to investigate the temporal expression dynamics. Synovial *S100a11* levels emerge as a promising diagnostic biomarker for rotator cuff tendinopathy, which is related to pain and dysfunction [37]. Adam8 inhibition represents a promising therapeutic approach via the regulation of neutrophil activity. Bioinformatics analyses further reveal associations between Adam8 and immune cell populations, including CD8+ T cells and resting memory CD4+ T cells, as well as its involvement in intervertebral disc degeneration [38]. Interestingly, Adam8 demonstrates protective effects in osteoarthritis by inhibiting fibroblast-like synoviocyte migration and invasion through the FSCN1/MAPK pathway [39]. Garman et al. [40] recently reported that upregulated Adam8 contributes to depressive-like behaviors in chronic pain conditions. Pmaip1, a pro-apoptotic protein, has been reported to be associated with the apoptotic induction of various cancer cells, such as spinal cord glioma cells [41,42], lung cancer cells [43] and mantle cell lymphoma [44]. Targeting Pmaip1-mediated apoptosis is considered an alternative anti-cancer strategy [45]. Ifit3, as a key member of the interferon-inducible protein family, exhibits vital roles in innate antiviral immunity and changes in cellular biology [46], including cell proliferation, apoptosis, and differentiation, as well as exacerbating inflammation-mediated tumor growth via interferon signaling, RIG-I-like receptors, and the NF-κB pathway [47]. Through bioinformatics analysis, *Ifit3* is also identified as a vital gene in pain-related diseases, such as dermatomyositis [48] and degenerative discs [49]. Cxcl10 has been well studied in NP pathogenesis [50,51,52,53], and *Cxcl10* showed an inverse pattern, displaying a burst upregulation at day 14. The expression of *Siglech*, *Mpeg1*, *Ly86*, *Crym*, *Cd180*, *Procr* and *Abi3* exhibited a progressive downregulated trend with NP pathogenesis. These results imply that *Siglech*, *Mpeg1*, *Ly86*, *Crym*, *Cd180*, *Procr* and *Abi3* may emerge as potential biomarkers in the acute phase, yet *Cxcl10* seems to be suitable as a chronic phase biomarker following peripheral nerve injury. The expression of *Adam8*, *Ifit3*, *Pld4*, *Pmaip1*, and *S100a11* remained stable without exhibiting time-dependent patterns, which suggests sustained activity on NP progression and indicates that they can be used as stable biomarkers.

Since the immune inflammatory response plays an essential role in NP pathogenesis, an immune infiltration analysis was performed based on 54 DEGs. The SNI group exhibits robust immune cell infiltration, dominated by B cell memory and especially monocytes, while the sham group exhibited a higher percentage of T cells CD8, T cells CD4 (memory resting), and macrophages M0. This distinct immune profile both confirms the pathogenic role of neuroinflammation in neuropathic pain and reveals actionable molecular targets for therapeutic intervention. Moreover, the Spearman correlation analysis revealed a positive correlation between monocytes and *S100a11*, *Gal*, *Ifit3*, *Ngfr*, *Coch*, *Pmaip1*, *Adam8*, *Mettl7a3*, *Fst*, *Cxcl10* and *Slc6a19*, which suggest the involvement of these genes in the monocytes’ infiltration of NP. Through a systematic literature search, we found that the expression of *S100a11* [54], *Gal* [55], *Ifit3* [56], *Ngfr* [57], *Adam8* [58], *Cxcl10* [59] in the monocytes has been well studied, while involvement in pain has not been elucidated yet except *Ngfr* [60] and *Cxcl10* [61].

Additionally, ROC analysis based on 11 DEGs confirmed the potential diagnostic value of *Gal*, *Pmaip1* and *Fst*. The construction of the TF–mRNA–miRNA network offers insights into candidate regulatory mechanisms and targeted therapeutics for NP. TFs regulate mRNA transcription by binding to specific sequences in gene promoter regions, thereby modulating gene expression. The regulatory interplay between TFs and mRNAs plays pivotal roles in diverse biological processes. Fourteen upstream TFs were predicted and might modulate *Pmaip1*, *Ngfr*, *Cxcl10*, *Slc6a19* and *Adam8* in the pathogenesis of NP. TFs, including Egr1 [62], Rela [63], Nfkb1 [64], Mitf [65] and Stat1 [66,67] have been reported in the NP, and their targeted pharmacological inhibition may represent a therapeutic strategy for NP. More explorations of TF-mRNA regulatory mechanisms are required to elucidate the regulation of these genes and develop novel therapeutic strategies for NP. MiRNAs are short, noncoding RNAs involved in the regulation of several processes associated with inflammatory processes. We categorize miRNAs derived from the 5′ and 3′ arms of the same precursor miRNA into one group, such as mmu-miR-7674-3p and mmu-miR-7674-5p. Thus, we selected a total of fourteen groups of miRNAs that can interact with more than five genes, which means that, once one group of miRNAs has been targeted, all five genes can be interfered with at the same time. However, the precise mechanisms underlying their roles in NP are not yet understood and require further study. NP-targeted drugs on 11 DEGs were also predicted in our study. Notably, six genes (*Cxcl10*, *Gal*, *Coch*, *Pmaip1*, *Ngfr* and *Fst*) have detectable proven drugs. Although only proven drugs were included in our study, the unproven drugs still need further investigation. The identification of target drugs uncovers potential mechanistic avenues for NP treatment.

There have been several studies on the bioinformatics analysis of neuropathic pain models. Korczeniewska et al. [68] performed trigeminal ganglia and DRG sequencing at day 4, day 8 and day 21 following infraorbital or sciatic nerve injuries but without WGCNA and machine learning analysis, as well as immune infiltration analysis. Another time-course analysis of NP research [69] focused on epigenetic changes in the m6A modification status of mRNA at the spinal cord level of SNI, elucidating its epigenetic regulatory role. Two recent studies have employed machine learning approaches to analyze GEO datasets, examining pyroptosis- [70] or anoikis-related DEGs [71] in NP. Our study takes advantages of combined bioinformatics and machine learning methods to analyze differential genes with time-series signatures in NP, which have not been verified before. Immune infiltration analysis contributes to our deeper understanding the involvement of immune cells in NP pathogenesis. We identified several key genes and biomarkers with temporal expression patterns, indicating that some genes may play important roles in the acute or chronic phase, while some exhibit sustained expression throughout the pathogenesis of neuropathic pain. These findings will provide new mechanistic insights into NP and offer the potential for developing alternative therapeutics or new diagnostic tools that may aid in the early identification and monitoring of pain states.

Nonetheless, this study has some limitations. First, the limited sample size constrained the performance of the machine learning models. To mitigate this, we employed simple models and repeated cross-validation. However, considering that cross-validation might further reduce the adequacy of the training data in a limited sample size, and that our initial analysis prioritized assessing model feasibility for screening characteristic genes over the final performance evaluation, we conducted cross-validation (with ‘nfold’ and ‘lambda’ in LASSO and SVM-RFE, and ‘ntree’ and ‘optionTrees’ in RF) without calculating accuracy, precision, or the F1-score. Thus, larger sample sizes for distinct time points are required to build prognostic models and conduct further analysis. The absence of external datasets was another limitation in our analysis. Future analysis will focus on and integrate external datasets with time courses that align with our research objectives for validation. Second, although mouse models share many similarities with human models, there still exist interspecies differences in gene expression and immune systems between humans and mice. To mitigate the impact of these potential differences on the extrapolation of our results, animal models were rigorously selected. The SNI model, which reproduces the pathophysiological features in clinical NP disorders, is considered a valuable tool for elucidating underlying molecular mechanisms [8,9]. We also undertook gene symbol matching between target genes and human immune cell genes, and only matches were allowed for further immune infiltration analysis. Despite these implemented measures, validation based on human samples is still required. Third, future in vivo and in vitro experiments are necessary to provide valuable insights. Although validation was conducted solely at the mRNA level via PCR analysis in the present study, complementary confirmation via Western blot analysis at the protein level or further experiments are lacking; these measures are necessary to confirm the functional roles of key genes and pathways. Future NP studies will require emerging external gene datasets and more experimental validation to gain deeper insights.

## 5. Conclusions

Overall, 54 potential DEGs were identified across three time points of NP and analyzed using multiple bioinformatics analysis and three machine learning algorithms. The subsequent integrative analysis prioritized 11 core genes via intersecting DEGs and WGCNA-derived feature module genes. Experimental validation via qRT-PCR confirmed mRNA-level consistency with computational predictions. These newly identified core genes exhibiting temporal expression patterns hold significant promise as potential therapeutic targets for NP treatment.

## Figures and Tables

**Table 1:** List of primer sequences for qRT-PCR analysis.

mRNA	Forward (5′–3′)	Reverse (5′–3′)
*Siglech*	GGAGAGACCAGCAACACACA	TCCAGTTGGCACCATCATCC
*Mpeg1*	CTGGATGATAATAGCGTGTGC	CAAGACAGGTAGTTTCAGGGC
*Ly86*	ATTCTGAACTACTCCTATCCCCTTT	GGCCGGCATAGTATATCTGTTCT
*Crym*	CTATGAGGGCCACAGCAACA	ATGACCGCCAGCAGGGAG
*Cd180*	TAGGTCTCAATGAAATTCCTGGC	AATCTGGCACCTGGTTAAATCC
*Procr*	TTGACGAAGTTTCTGCCGCTAC	CCTGATGCCTCACATGATGGTT
*Abi3*	CTACTGCGAGGATAACTACTTGC	CAGGTTACCCACTTGGTAGGC
*Cxcl10*	ATCCACCGCTGAGAGACATCCC	AATGACGGCAGCACTTGGGTTC
*Adam8*	GCAGGACCATTGCCTCTACC	TGGACCCAACTCGGAAAAAGC
*Ifit3*	ACTCCATCGTTAATCGTCTC	ACAGTGAACAACAGTCCTC
*Pld4*	TGGTGCCCAGATACGACA	AGGGATGGAAGCGGTTGA
*Pmaip1*	CTCAGGAAGATCGGAGACAAAGT	GAGTTGAGCACACTCGTCCTT
*S100a11*	AGCTGGACCTCAACTGT	GTAGGTGTGCTGGGCTC

qRT-PCR, quantitative real-time PCR.

## Data Availability

The raw data supporting the conclusions of this article will be made available by the authors on request.

## Supplementary Materials

The following supporting information can be downloaded at https://www.mdpi.com/article/10.3390/biom15091254/s1, Figure S1. The capture of spontaneous pain in mice. Figure S2. Hub genes in the PPI networks. Figure S3. Gene Ontology analysis on the whole gene set. Figure S4. Gene Ontology analysis on the whole gene set. Figure S5. Gene Ontology analysis on the whole gene set. Figure S6. Validation of DEGs expression by qRT-PCR. Figure S7. ROC analysis on the 11 DEGs. Table S1. 54 DEGs identified by integration three time-point groups. Table S2. GO and KEGG analysis on the 54 DEGs. Table S3. Genes identified by LASSO algorithms. Table S4. Genes identified by RF algorithms. Table S5. Genes identified by RVM-RFE algorithms.

## Abbreviations

The following abbreviations are used in this manuscript:

NP	Neuropathic pain
DEGs	Differentially expressed genes
GO	Gene ontology
KEGG	Kyoto Encyclopedia of Genes and Genomes
WGCNA	Weighted gene co-expression network analysis
LASSO	Least absolute shrinkage and selection operation
RF	Random forest
SVM-RFE	Support vector machine recursive feature elimination
qRT-PCR	Quantitative real time-PCR
ROC	Receiver operating characteristic
AUC	Area under the curve
SNI	Spared nerve injury
PUMCH	Peking Union Medical College Hospital
PPI	Protein–protein interaction
MDG	Mean decrease Gini
TOM	Topological overlap matrix
