# Supplementary material for: Biomarker Signatures in Time-Course Progression of Neuropathic Pain at Spinal Cord Level Based on Bioinformatics and Machine Learning Analysis
Source: Biomolecules. 2025 Aug 29;15(9):1254. doi: 10.3390/biom15091254 (PMC12467963; doi:10.3390/biom15091254)
Supplement: Supplementary file 1 [file biomolecules-15-01254-s001.zip › biomolecules-3738182-supplementary.pdf]

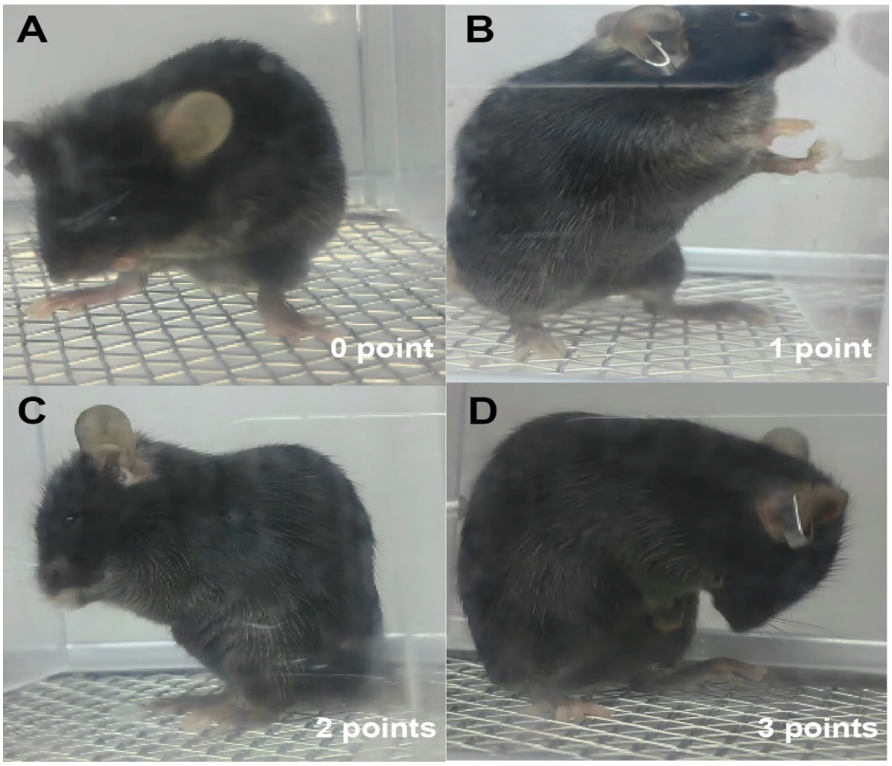


Supplementary Figure S1. The capture of spontaneous pain in mice. (A-D) Four kinds of spontaneous pain behaviors corresponding to spontaneous pain score: 0, 1, 2 and 3 points, n = 12 per group.

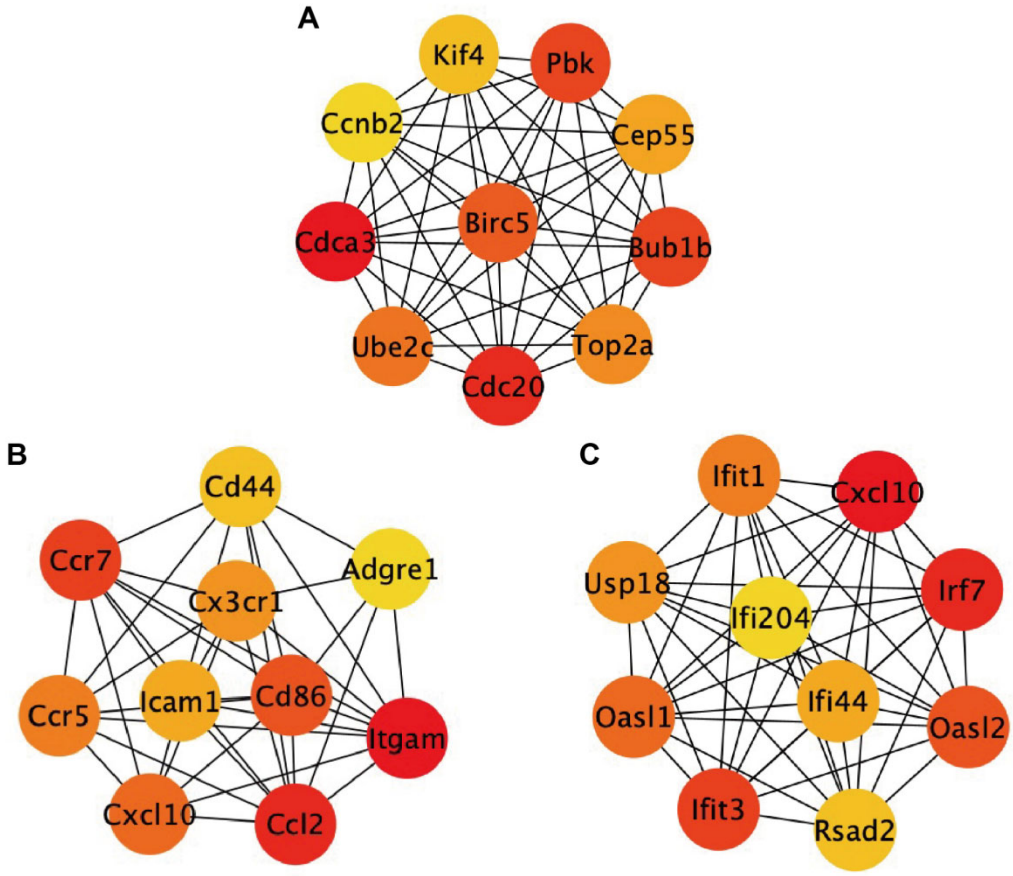


Supplementary Figure S2. Hub genes in the PPI networks. (A, B and C) Top ten genes were identified in the NP at day3, day7 and day14 respectively. The darker color of nodes represents the higher degree. PPI, protein-protein interaction; NP, neuropathic pain.

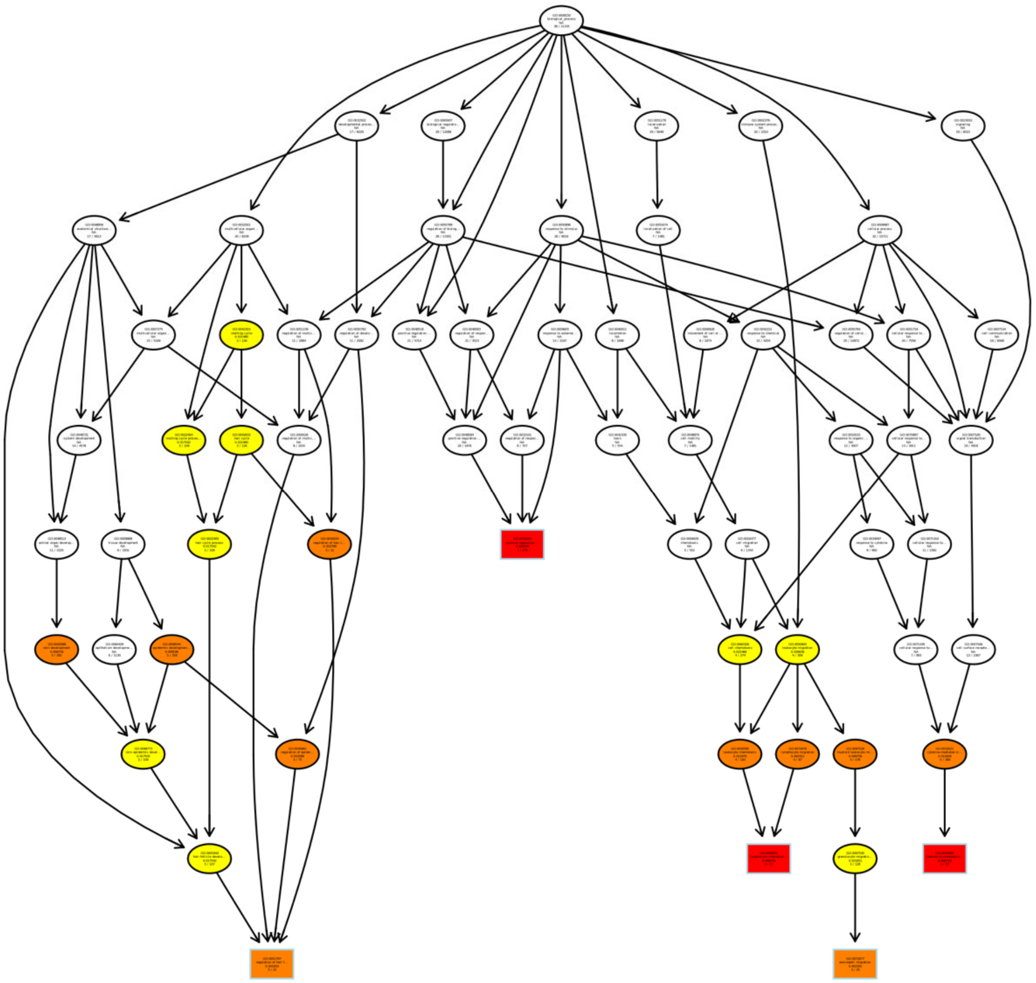

Supplementary Figure S3. Gene Ontology analysis on the whole gene set. Biological processes (BP) in the Gene Ontology analysis.

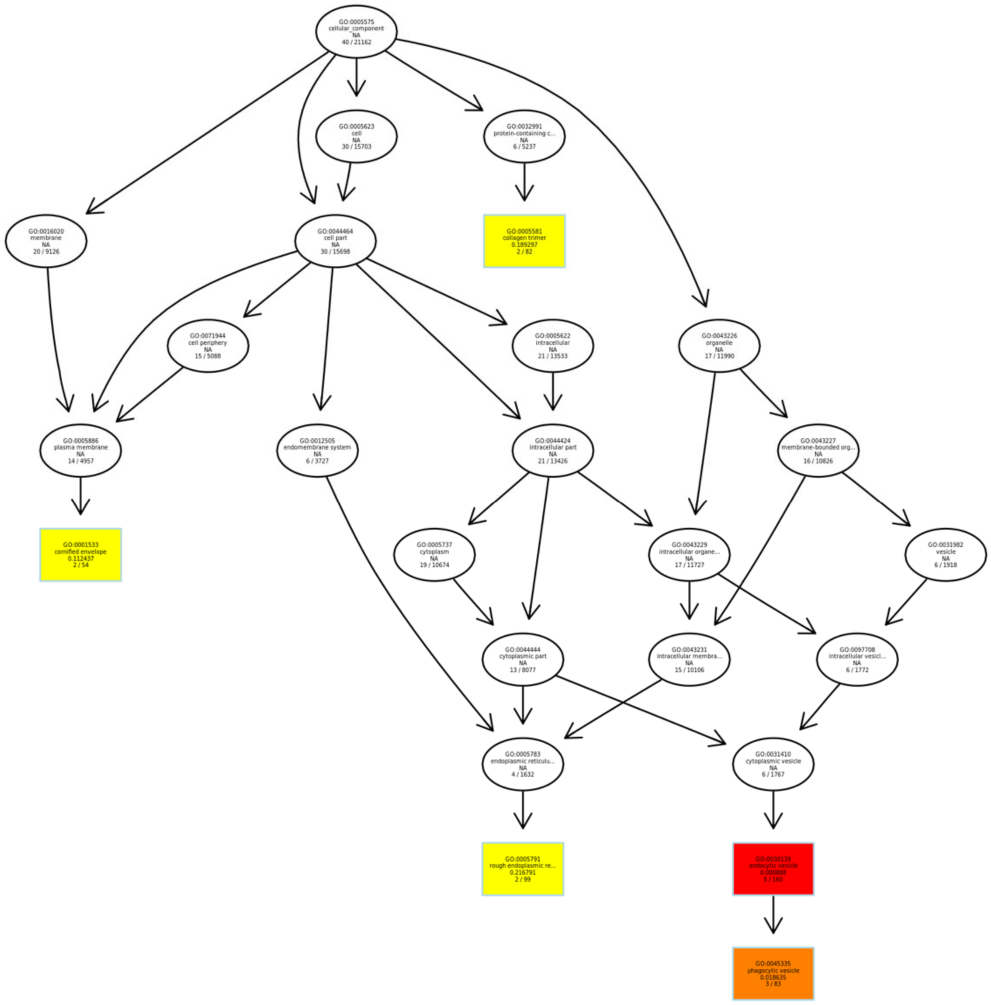


Supplementary Figure S4. Gene Ontology analysis on the whole gene set. Cellular components (CC) in the Gene Ontology analysis.

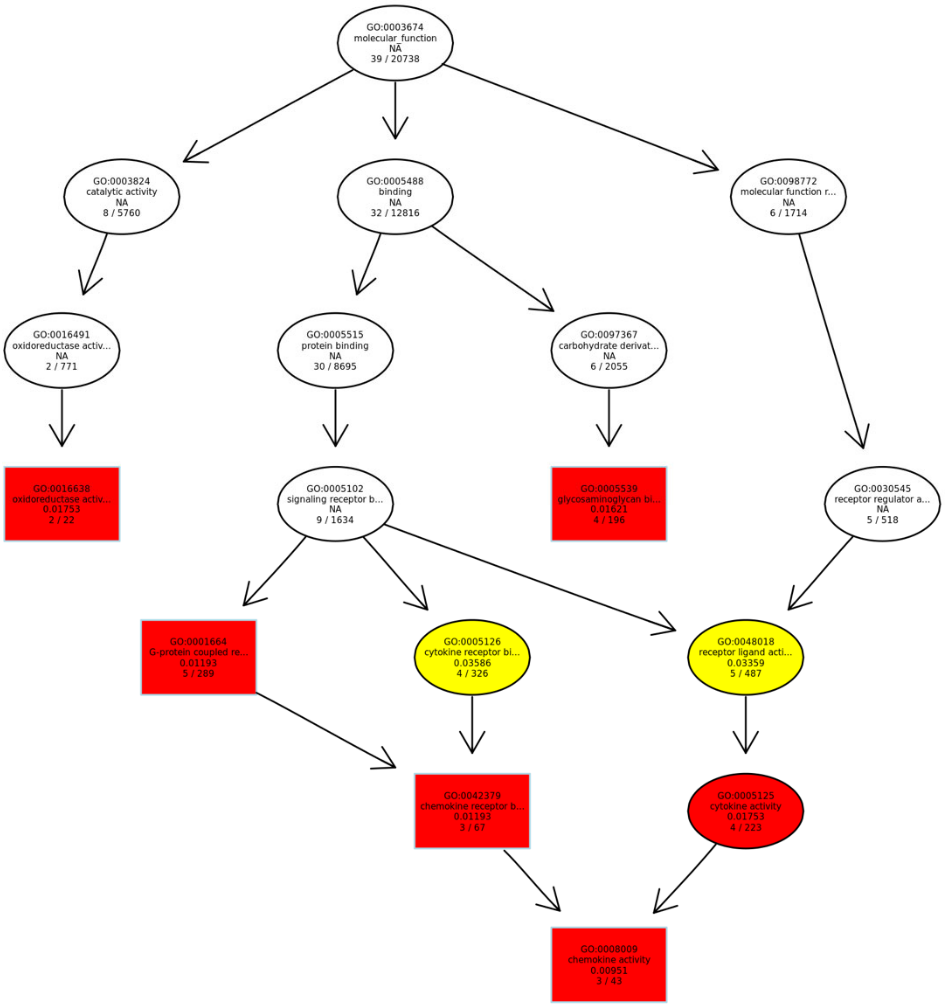


Supplementary Figure S5. Gene Ontology analysis on the whole gene set. Molecular functions (MF) in the Gene Ontology analysis.

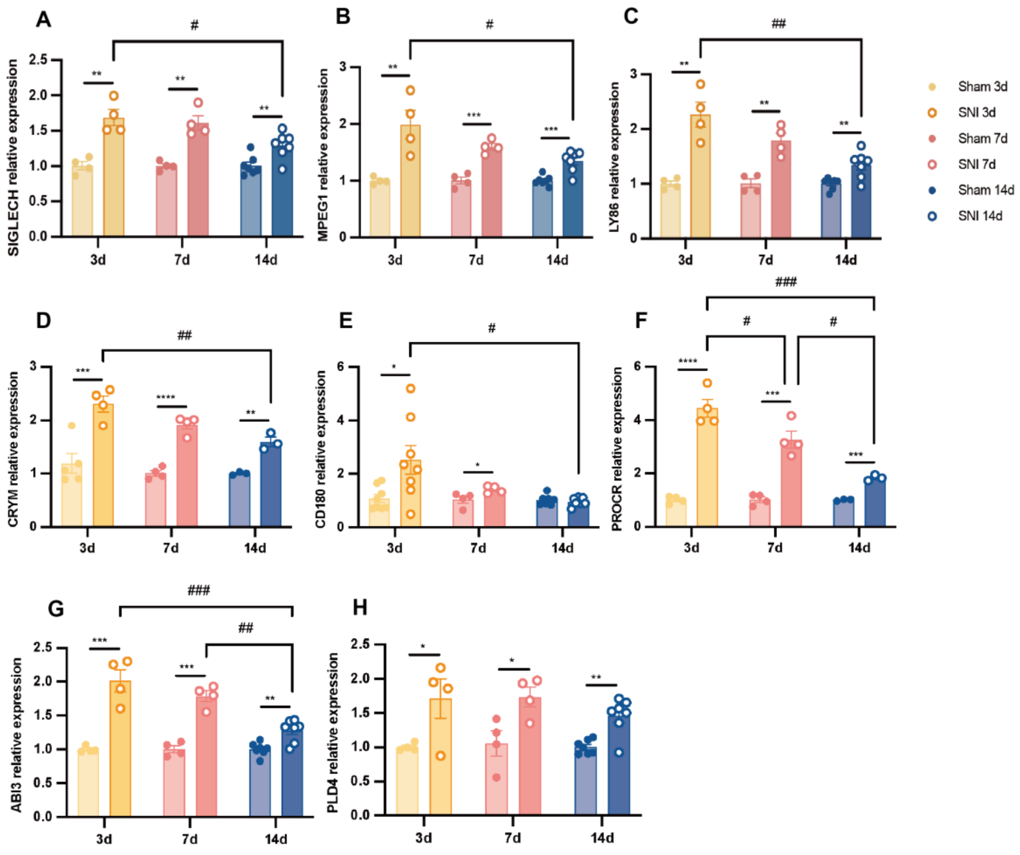


Supplementary Figure S6. Validation of DEGs expression by qRT-PCR. (A-H) Temporal changes of mRNA expression levels of Siglech, Mpeg1, Ly86, Crym, Cd180, Procr, Abi3 and Pld4, n = 4-8 per group. Data are represented as mean ± SEM. * represents comparison between SNI and sham at the same time point, while # represnts comparison between SNI groups at various time points. *, $p < 0.05$; **, $p < 0.01$; ***, $p < 0.001$; ****, $p < 0.0001$; #, $p < 0.05$; ##, $p < 0.01$, ###, $p < 0.001$.  DEGs, differentially expressed genes; qRT-PCR, quantitative real-time PCR.

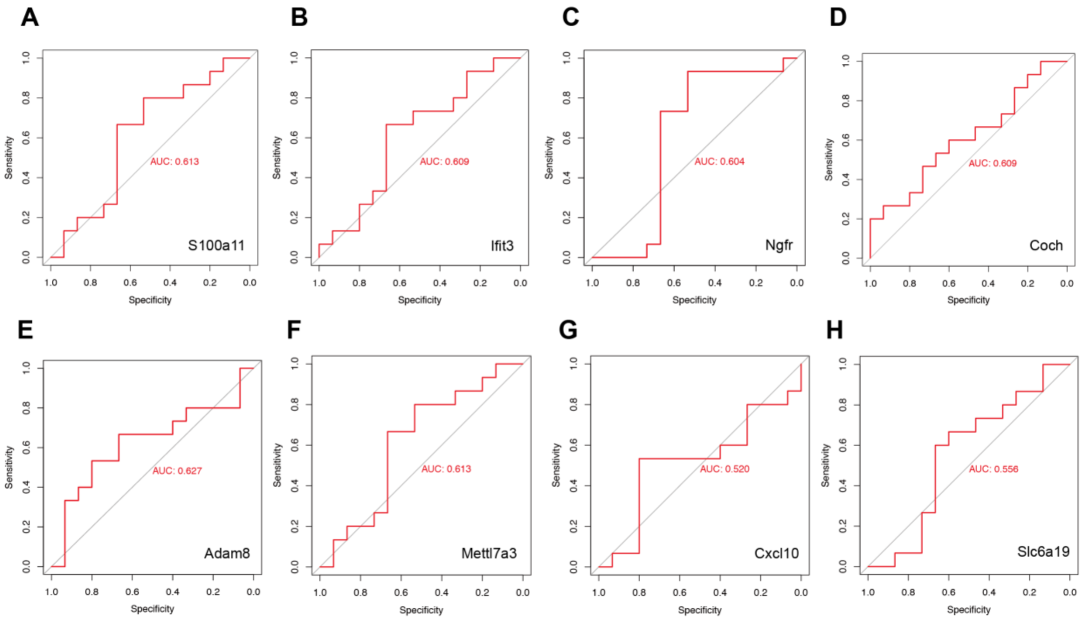


Supplementary Figure S7. ROC analysis on the 11 DEGs. The rest 8 genes were assessed with moderate diagnostic value (0.5 < AUC < 0.7). ROC, Receiver Operating Characteristic; AUC, Area Under the Curve; DEGs, differentially expressed genes.

**Supplementary Table S1. 54 DEGs identified by integration three time-point groups.**

| Gene | Gene | Gene | Gene |
|---|---|---|---|
| Ctss | Fcgr1 | Gm5152 | F2rl1 |
| C1qa | Coch | Gm26945 | 4833415N18Rik |
| Sprr1a | Procr | Lce1g | Gm21178 |
| C1qc | Gpr151 | Fcgr4 | Liph |
| S100a11 | Pmaip1 | Loxl4 | Ankrd1 |
| Gal | Kcnk6 | Ccl2 | Prokr2 |
| Mpeg1 | Adam8 | Abi3 | Lipk |
| Atf3 | Anxa10 | Gm47903 | Gm31831 |
| Ifit3 | Ccl7 | Crym | Rmi2 |
| Pld4 | Mettl7a3 | Fst | 4932442E05Rik |
| Siglech | Capn3 | Cxcl10 | Gm10354 |
| Ly86 | Timp1 | Slc6a19 | Gm5084 |
| Ngfr | Was | Gm35569 | Sh2d1b2 |
| Trem2 | Gm15344 | | |

DEGs, differentially expressed genes.

**Supplementary Table S2. GO and KEGG analysis on the 54 DEGs.**

| Category | ID | Description | Padj | Genes |
|---|---|---|---|---|
| BP | GO:0032103 | Positive regulation of response to external stimulus | 0.0005 | Ctss/Ly86/Fcgr1/Adam8/Cd180/Ccl2/Cxcl10 |
| | GO:0048247 | Lymphocyte chemotaxis | 0.0007 | Adam8/Ccl7/Ccl2/Cxcl10 |
| | GO:0070098 | Chemokine-mediated signaling pathway | 0.0007 | Trem2/Ccl7/Ccl2/Cxcl10 |
| | GO:0072677 | Eosinophil migration | 0.0023 | Adam8/Ccl7/Ccl2 |
| | GO:0031349 | Positive regulation of defense response | 0.0024 | Ctss/Fcgr1/Coch/Adam8/Cd180/Sh2d1b2 |
| | GO:0072676 | Lymphocyte migration | 0.0024 | Adam8/Ccl7/Ccl2/Cxcl10 |
| | GO:0019882 | Antigen processing and presentation | 0.0028 | Ctss/Trem2/Fcgr1/Was |
| | GO:0032496 | Response to lipopolysaccharide | 0.0030 | Ly86/Trem2/Cd180/Ccl2/Cxcl10 |
| CC | GO:0030139 | Endocytic vesicle | 0.0009 | Pld4/Ngfr/Adam8/Was/Ccl2 |
| | GO:0045335 | Phagocytic vesicle | 0.0186 | Pld4/Adam8/Was |
| MF | GO:0008009 | Chemokine activity | 0.0095 | Ccl7/Ccl2/Cxcl10 |
| | GO:0001664 | G-protein coupled receptor binding | 0.0119 | Gal/Fcgr1/Ccl7/Ccl2/Cxcl10 |
| | GO:0042379 | Chemokine receptor binding | 0.0119 | Ccl7/Ccl2/Cxcl10 |
| | GO:0005539 | Glycosaminoglycan binding | 0.0162 | Trem2/Ccl7/Ccl2/Cxcl10 |
| | GO:0016638 | oxidoreductase activity, Acting on the CH-NH2 group of donors | 0.0175 | Loxl4/Crym |
| | GO:0005125 | Cytokine activity | 0.0175 | Ccl7/Timp1/Ccl2/Cxcl10 |
| | GO:1901681 | Sulfur compound binding | 0.0188 | Ccl7/Ccl2/Fst/Cxcl10 |
| | GO:0048018 | Receptor ligand activity | 0.0336 | Gal/Ccl7/Timp1/Ccl2/Cxcl10 |
| | GO:0043394 | Proteoglycan binding | 0.0336 | Ctss/Fst |
| | GO:0005126 | Cytokine receptor binding | 0.0359 | Ngfr/Ccl7/Ccl2/Cxcl10 |
| KEGG | mmu04061 | Viral protein interaction with cytokine and cytokine receptor | 0.0104 | Ccl7/Ccl2/Cxcl10 |
| | mmu04062 | Chemokine signaling pathway | 0.0104 | Ccl7/Was/Ccl2/Cxcl10 |
| | mmu04610 | Complement and coagulation cascades | 0.0104 | C1qa/C1qc/Procr |
| | mmu04060 | Cytokine-cytokine receptor interaction | 0.0118 | Ngfr/Ccl7/Ccl2/Cxcl10 |
| | mmu04657 | IL-17 signaling pathway | 0.0167 | Ccl7/Ccl2/Cxcl10 |

| | | | |
|---|---|---|---|
| mmu04148 | Efferocytosis | 0.0255 | C1qa/C1qc/Ano7 |
| mmu04215 | Apoptosis - multiple species | 0.0280 | Ngfr/Pmaip1 |

GO, Gene Ontology; KEGG, Kyoto Encyclopedia of Genes and Genomes; DEGs, differentially expressed genes; BP, biological processes; CC, cellular components; MF, molecular functions.

**Supplementary Table S3. Genes identified by LASSO algorithms.**

| Gene | Coef |
|---|---|
| Siglech | -1.388859392 |
| Ly86 | -1.394098815 |
| Ngfr | 0.676544603744839 |
| Kcnk6 | -2.215781316 |
| Lce1g | 0.00424064044598425 |
| Abi3 | -1.077882444 |
| Cxcl10 | 2.90004219640473 |
| Slc6a19 | 2.86666155644596 |
| Gm35569 | -11.17811754 |
| Ankrd1 | -0.168034175 |
| Prokr2 | -3.505862284 |
| Lipk | 6.0358947861455 |
| Gm5084 | -15.79215657 |

**Supplementary Table S4. Genes identified by RF algorithms.**

| Gene | Importance | Gene | Importance |
|---|---|---|---|
| Ly86 | 1.13273131380717 | Mpeg1 | 0.205381944444444 |
| Kcnk6 | 0.901210982644806 | Liph | 0.189246286121286 |
| Gm35569 | 0.891299004666383 | Pld4 | 0.184469696969697 |
| Siglech | 0.761557807975142 | Gm15344 | 0.182655234099742 |

| | | | |
|---|---|---|---|
| Ngfr | 0.75486324731197 | Was | 0.178475268031189 |
| Procr | 0.657205262028631 | Slc6a19 | 0.170860042735043 |
| Gm5084 | 0.526300955988456 | Gpr151 | 0.170773517740429 |
| Abi3 | 0.515711862181357 | X4932442E05Rik | 0.157208994708995 |
| Ccl2 | 0.489399477228424 | Ccl7 | 0.155216640085061 |
| Capn3 | 0.469572230601642 | F2rl1 | 0.149842923280423 |
| Timp1 | 0.434250992063492 | Lce1g | 0.148595328282828 |
| Ankrd1 | 0.404222463839337 | Sprr1a | 0.127314814814815 |
| Ctss | 0.365524765195818 | Gm21178 | 0.118002136752137 |
| X4833415N18Rik | 0.359023031062505 | Fcgr1 | 0.106517094017094 |
| Crym | 0.354112973749701 | Prokr2 | 0.102380952380952 |
| Gm47903 | 0.332462566673093 | Sh2d1b2 | 0.09375 |
| Adam8 | 0.317207159312422 | Anxa10 | 0.0895833333333333 |
| Fst | 0.308748287531182 | Atf3 | 0.0783730158730159 |
| Rmi2 | 0.258627471456419 | Gal | 0.0590277777777778 |
| C1qc | 0.256324404761905 | Ifit3 | 0.0573177426438295 |
| Cxcl10 | 0.252660516914622 | Trem2 | 0.05625 |
| Pmaip1 | 0.250720838316746 | Lipk | 0.0424077733860342 |
| Mettl7a3 | 0.229267219708396 | S100a11 | 0.0388888888888889 |
| Coch | 0.225267094017094 | Fcgr4 | 0.03125 |
| C1qa | 0.21210533126294 | Mpeg1 | 0.205381944444444 |

RF, Random Forest.

**Supplementary Table S5. Genes identified by RVM-RFE algorithms.**

| **Gene** |
|---|
| Gm31831 |

| |
|---|
| Atf3 |
| Ctss |
| Gpr151 |
| Ankrd1 |
| Adam8 |
| Trem2 |
| Lce1g |
| Mpeg1 |
| C1qc |
| C1qa |
| Ngfr |
| Sprr1a |
| Pld4 |
| Sh2d1b2 |

RVM-RFE, Support Vector Machine - Recursive Feature Elimination.
